# Evaluation of the rhizospheric microbiome of the native colonizer *Piptatherum miliaceum* in semiarid mine tailings

**DOI:** 10.1007/s10653-022-01357-y

**Published:** 2022-09-08

**Authors:** Héctor M. Conesa, Isabel Párraga-Aguado, Francisco J. Jiménez-Cárceles, Yolanda Risueño

**Affiliations:** 1 Departamento de Ingeniería Agronómica, Escuela Técnica Superior de Ingeniería Agronómica, Universidad Politécnica de Cartagena, Paseo Alfonso XIII, 48, 30203 Cartagena, Spain; 2 Cartagena, Spain; 3 BIOCYMA, Consultora en Medio Ambiente y Calidad, S.L., C. Azarbe del Papel, 10, 30007 Murcia, Spain

**Keywords:** Mining contamination, Phytostabilization, Semiarid climate, Rhizosphere, Metals

## Abstract

The study of the rhizospheric microbiome in native plants should be a prerequisite before carrying out the phytomanagement of mine tailings. The goal of this work was to evaluate the rhizospheric microbiome of *Piptatherum miliaceum* in semiarid mine tailings. A comprehensive edaphic characterization was performed including the description of soil microbial composition in the rhizosphere of *P. miliaceum* growing at a mine tailings pile and at a control site. Plant nutritional and isotopic compositions were also determined. Neutral pH of the tailings (7.3) determined low metal extractability in 0.01 M CaCl_2_ (e.g. < 1 mg/kg for Zn). In spite of the contrasting edaphic fertility conditions of both sites, N (~ 15 g kg^−1^) and P (~ 400 mg kg^−1^) leaf concentrations were similar. The lower δ^15^N at the tailings plants (− 4.50‰) compared to the control (6.42‰) indicated greater efficiency of *P. miliaceum* for uptaking N under the low fertility conditions of the tailings (0.1% total soil nitrogen). The presence at the tailings of bacterial orders related to the cycling of N, such as *Rhizobiales,* could have contributed to enhance N acquisition. The lower leaf δ^13^C values at the tailings (− 30.22‰) compared to the control (− 28.47‰) indicated lower water use efficiency of the tailing plants. Some organotrophic bacterial and fungal groups in the tailings’ rhizospheres were also found in the control site (e.g.* Cytophagales*, *Sphingobacteriales* for bacteria; *Hypocreales*, *Pleosporales* for fungi). This may indicate that *P. miliaceum* is able to shape its own specific microbiome at the tailings independently from the initial microbial composition of the tailings.

**Supplementary Information:**

The online version contains supplementary material available at 10.1007/s10653-022-01357-y.

## Introduction

Mine tailings piles are formed by the leftover of the refining process of metal sulphide mineral ores. These structures may pose serious environmental risks because enriched metal particles can be spread by erosion from their bare surfaces (Conesa & Schulin, 2010). The phytomanagement through phytostabilization of semiarid mine tailings is considered a suitable technique to reduce their environmental risks (Álvarez-Rogel et al., 2021). This technique consists of introducing a stable vegetation cover at the tailings’ surfaces to diminish erosion and control metal fluxes through the soil profile (Domínguez et al., 2008). The key factor to achieve a suitable performance of vegetation in tailings lays on introducing plant species that are adapted to their unfavourable edaphic conditions, which may include, among others, high metal concentrations, high salinity, low nutrient availability or extreme pHs (Álvarez-Rogel et al., 2021). Thus, the criteria for selecting suitable plant species must include the tolerance to those edaphic conditions, but also, a strong root system to attach soil particles, low metal uptake to avoid risks into the food chain and, in the case of semiarid areas, adaptation to drought (Clemente et al., 2012).

Traditionally, most of studies on the phytomanagement of mine tailings have been usually based on plant metal uptake capabilities or the improvement of edaphic properties by soil amendments, obviating the understanding of the soil–plant interactions which may trigger edaphic successional processes (Párraga-Aguado et al., 2014). New approaches to the phytomanagement of semiarid tailings also suggest the need of evaluating the recovery of edaphic functionality provided by spontaneous plant colonizers to assure the long-term stability of the restored ecosystem, above all in limited resource systems such as those found in semiarid areas (Álvarez-Rogel et al., 2021; Peñalver-Alcalá et al., 2021). In this way, the biogeochemical processes induced by plant rhizospheres may trigger edaphic successional changes which, in turn, may support a self-sustaining and robust vegetation cover (Párraga-Aguado et al., 2013; Álvarez-Rogel et al., 2002). This mechanism of plant facilitation has been usually obviated as a feasible issue in the restoration of degraded ecosystems worldwide (Gómez-Aparicio, 2009), but it is of critical importance in semiarid tailings with low levels of soil organic matter and water availability (Álvarez-Rogel et al., 2022). A key factor to evaluate the potential role of a plant species to support soil biogeochemical processes at mine tailings lays on the description of the rhizosphere-mediated processes, and especially, of the specific microbiome associated with those native plants which colonize abandoned mine tailings (Xiao et al., 2019). The benefits generated by the autochthonous plant growth promoting bacteria of pioneer colonizer vegetation on sustaining soil nutrient cycles and on plant nutrient acquisition may even decrease the need of soil conditioners, which are usually used in phytostabilization (Grandlic et al., 2008). Soil rhizospheric microbes can interact with plants at tailings, decreasing their environmental stress (e.g. metals, salinity) and facilitating nutrient acquisition (Xiao et al., 2019). The heterogeneity of mine tailings’ surfaces generates biogeochemical gradients, which are not only responsible for determining plant colonization (Párraga-Aguado et al., 2013) and microbial composition but also the metabolic potential in the cycling of organic matter (Sun et al., 2020). However, most of the studies on the phytomanagement of tailings have usually focused on evaluating plant tolerance to adverse edaphic factors, obviating aspects related to the potential of spontaneous edaphic successional processes in the improvement of soil functionality in these multi-stressed ecosystems (Álvarez-Rogel et al., 2021). Thus, the study of the specific rhizosphere microbiome in native plants which grow at semiarid tailings and its potential link to plant ecophysiological and nutritional status will be considered in this work.

The goal of this work was to evaluate the rhizospheric microbiome of the native pioneer colonizer *Piptatherum miliaceum* in semiarid mine tailings and its potential role to facilitate plant growth and survival in these stressed ecosystems. For this purpose, a comprehensive edaphic characterization was performed including the description of the soil microbial composition (bacteria and fungi) of the rhizosphere of *P. miliaceum* growing at a mine tailings pile and at an abandoned dry crop land (taken as a control). In addition, plant leaf nutritional and isotopic composition were also determined at each sampling site.

## Material and methods

### Study site

**Fig. 1:**
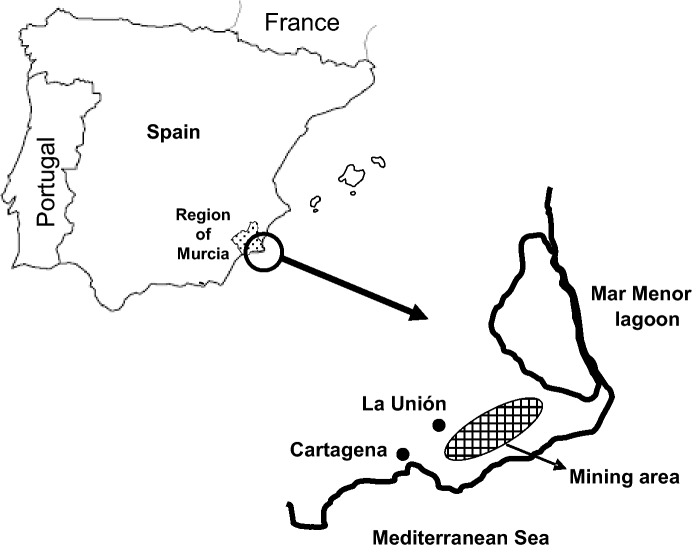
Location of the Cartagena-La Unión Mining District

The sampling site was located at a mine tailings pile in the semiarid Cartagena-La Unión mining district (southeast Spain, 110–0 m, a.s.l.; 37°37′20″ N, 0°50′55″ W–37°40′03″ N, 0°48′12″ W, Fig. 1 and SM1). Annual average temperature is 18 °C, while annual rainfall is 250–300 mm. Mining activity was focused on metallic sulphide minerals such as galena, pyrite and sphalerite. Additional information about the consequences of mining in this region was reviewed by Conesa and Schulin (2010).

### Selected plant species and sampling

The Gramineae *Piptatherum miliaceum* (L.) Coss. subsp. *miliaceum* was selected for study. This plant species behaves as an opportunist ruderal plant species and normally colonizes abandoned crop areas or anthropic altered sites (Martínez-Oró et al., 2017). It has a strong root system and robust rhizomes, which may protect soil against erosion (De Baets et al., 2007). This plant species has been previously suggested to act as a potential nurse species which may facilitate the establishment of late successional plant species at tailings (Morugán-Coronado et al., 2021).

A mine tailings pile (Figure SM2) and an abandoned dry crop land field (taken as a control site, Figure SM3) with the presence of *P. miliaceum* were selected like sampling sites. At the mine tailings pile, bare and *P. miliaceum* rhizosphere soil samples were taken from the first 30 cm of top soil. At the control site, only rhizospheric samples of *P. miliaceum* were taken because there were no bare spots free of vegetation. Rhizospheric samples consisted of the volume of soil surrounding few centimetres from the root epidermis (Samad et al., 2019). Three replicates were taken for all the cases. An aliquot of each sample was stored in sterile Falcon tubes, kept in ice in field and stored at − 20 °C till enzymatic and microbial analysis were performed. Soil samples for edaphic characterization were air-dried at room temperature, sieved through 2 mm, homogenized and stored in sealed plastic bags prior to laboratory analytical procedures.

### Soil analyses

A comprehensive soil characterization was performed. A 1:5 soil/water ratio extraction was used to measure pH, electrical conductivity (EC), water-extractable ions (Metrohm Ion Chromatographer for Cl^−^, SO_4_^2−^, Ca^2+^, K^+^, Mg^2+^, Na^+^) and dissolved organic carbon (TOC-VCSH Shimadzu). Readily extractable metal (Cu, Mn, Pb, Zn) concentrations were assessed through a 1:2.5 soil/solution ratio (g:ml) extracts using 0.01MCaCl_2_ (Sigma-Aldrich) as a reagent (González et al., 2011) and using an ICP-MS (Agilent 7500A). In ground soil samples, total nitrogen was determined following the Kjeldahl method (USDA, 1996), and organic carbon, after oxidising organic matter with potassium dichromate (Duchaufour, 1970). Total element concentrations were determined by X-ray fluorescence (Bruker S4 Pioneer). Particle size distribution was measured according to the method of Bouyoucos’ densimeter (Gee & Bauder, 1986). The percentage of equivalent calcium carbonate was determined following the Bernard calcimeter method (Hulseman, 1966; Muller & Gastner, 1971). Soil enzymatic activities that included dehydrogenase (García et al., 1993) and β-glucosidase (Reboreda & Caçador, 2008) were measured in unaltered portions of soil samples stored at − 20 °C. Microbial biomass carbon (MBC) was determined by the measurement of the extractable organic carbon by 0.5 M K_2_SO_4_ (Sigma-Aldrich) after a 24 h CHCl_3_-fumigation (Vance et al., 1987; Wu et al., 1990) employing a TOC-automatic analyser (TOC-VCSH Shimadzu). Available phosphorus (available-P) was measured following the Olsen method (Olsen et al., 1954) after using 0.5 M NaHCO_3_ (Sigma-Aldrich) solution at pH 8.5 as extractant and measuring the extracted PO_4_^3−^ by a Lambda 25 UV/VIS spectrometer (PerkinElmer) at λ = 820 nm.

Microbial (bacteria and fungi) DNA was extracted from 0.25 g soil samples using the PowerSoil DNA Isolation Kit (MOBIO), according to the manufacturer’s instructions. The isolated DNA was quantified using a NanoDrop 2000 spectrophotometer. Library preparation and Illumina sequencing were carried out at the IPBLN Genomics Facility (CSIC, Granada, Spain). Raw sequence data in FASTQ format (16S and ITS2) were subjected to quality control analysis with FastQC software and prepared for taxonomic classification using the Mothur software (version 1.43.0) (Schloss et al., 2009) and following the standard operating protocol proposed by Kozich et al. (2013). Phyla and orders (both bacteria and fungi) that showed > 5% relative abundance in at least one sampling site were considered. The taxa with < 0.5% of relative abundance were discarded for comparison. Extended protocols for sequencing and bioinformatics are available at the Supplementary Material.

### Plant analyses

At each location, plant leaf samples were taken (three replicates). Plant samples for elemental and stable isotope analyses were treated as it was described in Párraga-Aguado et al. (2014). Leaves were carefully washed with distilled water and dried at 65 °C for 72 h prior to grinding. For each sample, 0.1–0.5 g was incinerated prior to a redilution using concentrated nitric acid. The resulting extracts were filled to 25 ml using deionized water and filtered through CHM F2041-110 ashless filter papers (20–25 μm pore diameter). Then, metals (Cu, Mn, Pb, Zn) were analysed using an ICP-MS (Agilent 7500A), and P and S were analysed using an Ion Chromatographer (Metrohm). Plant analyses were referenced using a CTA-VTL-2 certified material (Virginia tobacco leaves). The percentage of recoveries for ranged from 96 to 108%.

Finely ground plant material was used for stable isotope measurements at the University of California-Davis Stable Isotope Facility. Leaf C, δ^13^C, N and δ^15^N analyses were conducted using a PDZ Europa ANCA-GSL elemental analyser interfaced to a PDZ Europa 20-20 isotope ratio mass spectrometer (Sercon Ltd., Cheshire, UK). δ^13^C and δ^15^N data are expressed relative to international standards V-PDB (Vienna PeeDee Belemnite).

### Statistics

Statistical analyses were performed using the IBM SPSS Statistics 24 software. Homogeneity of variances was tested by using the Levene’s test. Data non-normally distributed were log-transformed to fit to a normal distribution prior to statistical analyses. T-test was employed for comparing two groups.

## Results and discussion

### Edaphic parameters

The mine tailings samples (bare and *P. miliaceum*’s rhizosphere) showed neutral pH values, while control samples were slightly alkaline (Table 1). In relation to the electrical conductivity (EC) values, the tailings samples were considered as extremely saline (EC > 2 dS m^−1^) (Alarcón-Vera, 2004). Although the samples from the tailings pile (both, bare and rhizosphere) and the control area showed similar values of EC (*p* > 0.05), their salt composition differed: in the tailings samples the main contributor to EC was SO_4_^2−^ (8700 mg kg^−1^) whose concentrations were 18-fold higher than at the control site. Moreover, Cl^−^ and SO_4_^2−^ showed similar concentrations (327 mg kg^−1^ and 476 mg kg^−1^, respectively) in the control samples. The high SO_4_^2−^ and Ca^2+^ water-extractable concentrations of the tailings samples could be explained by the presence of gypsum, which is secondary formed in neutral pH mine tailings (García-Lorenzo et al., 2012). No differences of total metal concentrations were found between the bare and *P. miliaceum* rhizosphere tailings samples (Table 1). A comprehensive study of the geochemical backgrounds of Murcia Region done by Martínez-Sánchez and Pérez-Sirvent (2007) showed that the soils from the nearby Cartagena plain had a geochemical background of 12.6 mg kg^−1^ Cu, 9 mg kg^−1^ Pb and 41 mg kg^−1^ Zn total concentrations. These authors considered that the thresholds in the soils from the Cartagena-La Union mining area should be established separately due to their different geochemical basis. For comparing, it is usually meaningful to employ technical reports or studies carried out on local soils. Following this methodology, some agricultural soils located around La Union town have shown total concentrations of 21 mg kg^−1^ Cu, 500 mg kg^−1^ Pb and 900 mg kg^−1^ Zn (Conesa et al., 2010) which are in the range of our control site. In addition, total metal concentrations at our tailings site are also in the range of other studies carried out at local tailings (García-Lorenzo et al., 2012; Peñalver-Alcalá et al., 2021).

**Table 1:** Characterization of soil samples

Soil parameters	Units	Tailings pile				Control
		Bare mine tailings		Rhizosphere at tailings		Rhizosphere at not impacted site
pH		7.31 ± 0.12		7.18 ± 0.02	*	7.85 ± 0.01
%CaCO_3_	%	5.06 ± 0.23		4.48 ± 1.11	*	14.83 ± 0.48
Electrical conductivity	dS m^−1^	2.78 ± 0.01		2.90 ± 0.07		1.96 ± 0.36
Organic Carbon	g kg^−1^	2.0 ± 0.3		3.0 ± 0.5	*	20.6 ± 1.2
Dissolved Organic Carbon	mg kg^−1^	21.3 ± 1.9 *		36.1 ± 1.9	*	386.3 ± 63.8
Total Nitrogen	g kg^−1^	0.10 ± < 0.01		0.17 ± 0.04	*	3.86 ± 1.14
δ^15^N	‰	− 1.49 ± 0.57 *		0.70 ± 0.38	*	11.79 ± 0.76
Available P	mg kg^−1^	5.7 ± 1.1 *		12.5 ± 0.4	*	145.7 ± 9.0
*Microbiological parameters*						
Microbial Biomass Carbon	mg kg^−1^	32.1 ± 3.8		52.5 ± 1.4	*	394.6 ± 12.7
Dehydrogenase	µg INTF g^−1^ h^−1^	0.18 ± 0.04 *		0.60 ± 0.05	*	5.34 ± 0.44
β-glucosidase	µmol p-NP g^−1^ h^−1^	0.12 ± 0.01 *		0.38 ± 0.07	*	2.52 ± 0.60
*Particle size distribution*						
clay	%	1.0 ± < 0.1		2.2 ± 1.2		20.8 ± 1.8
silt		4.1 ± 2.0 *		17.8 ± 0.9		38.0 ± 4.3
sand		94.9 ± 2.0 *		80.0 ± 2.1		41.1 ± 3.2
*Water-extractable ions*						
Cl^−^	mg kg^−1^	17.2 ± 5.8 *		63.7 ± 11.2	*	476.1 ± 44.5
SO_4_^2−^		8676 ± 19 *		8724 ± 97	*	691 ± 121
Na^+^		8.7 ± 0.5 *		60.0 ± 3.9	*	248.5 ± 57.2
K^+^		20.7 ± 7.1		51.7 ± 11.8	*	671.6 ± 153.4
Ca^2+^		2951 ± 17		3048 ± 35	*	382 ± 44
Mg^2+^		142.9 ± 3.9 *		361.7 ± 23.2	*	87.3 ± 1.0
*Total metal concentrations*						
Cu	mg kg^−1^	125 ± 5		120 ± 6	*	47 ± 15
Mn		9838 ± 94		10,537 ± 1482	*	560 ± 52
Pb		6518 ± 162		7382 ± 364	*	611 ± 61
Zn		9940 ± 110		9028 ± 463	*	462 ± 100
*0.01 M CaCl*_*2*_*-extractable concentrations*						
Cu	µg kg^−1^	78 ± 6 *		12 ± 2	*	97 ± 10
Mn		120 ± 4 *		52 ± 10	*	663 ± 105
Pb		24 ± 7		< 10		20 ± 6
Zn		921 ± 53		766 ± 136	*	211 ± 23

Data are average ± standard error. Number of replicates is three. Parameters with 1:5 were measured in the 1(soil):5(water) extract. Available-P is the phosphorus measured through the Olsen method

*Between columns represents significant differences (*t*-test, *p* < 0.05) in relation to the tailings rhizosphere samples

The neutral pH of the tailings may have also determined low 0.01 M CaCl_2_-extractable concentrations (Risueño et al., 2020). For some metals, such as Cu and Mn, the 0.01 M CaCl_2_ extractable concentrations were even higher at the control site (but not in phytotoxic levels) than at the mine tailings rhizospheres, indicating the determinant role of other edaphic parameters such as organic matter or texture on metal availability. This also indicated the secondary role that metal concentrations may play for the establishment of plants at neutral pH tailings. As it has been stated by previous authors, other parameters different from metal concentrations are determinant for plant colonization at neutral pH tailings, including particle size distribution or salinity (Párraga-Aguado et al., 2013; Risueño et al., 2020). In our study, the rhizospheres of *P. miliaceum* at the tailings pile showed higher silt and lower sand percentages than the bare tailings samples. It is likely that this slight modifications in edaphic parameters at tailings might be enough to trigger successional ecological processes and facilitate the establishment of pioneer plant species (Párraga-Aguado et al., 2014). The enhancement of soil fertility properties induced by the presence of *P. miliaceum* at the tailings was shown in the higher values (*p* < 0.05) of dissolved Organic Carbon (DOC), soil δ^15^N and available-P of the rhizospheric tailings samples in relation to the bare ones. In turn, this could have promoted higher microbiological activity within the rhizosphere as it was corroborated by the higher values (*p* < 0.05) obtained in the tailings rhizospheric samples for microbial biomass carbon (MBC), dehydrogenase and β-glucosidase. The control samples showed better soil fertility and microbiological indicators (*p* < 0.05) than the rhizosphere tailings samples.

### Plant elemental and isotopic composition

No differences were obtained for C, N and P concentrations when comparing leaf analysis between the tailings and the control site (Table 2). However, S and metal concentrations were higher in the plants from the tailings. Nitrogen and P are known to be limiting factors for plant growth in Mediterranean ecosystems (Martínez-Oró et al., 2017). The similar elemental composition for these two nutrients between the plants of our study coming from two different locations with contrasting fertility properties revealed the ruderal behaviour of *P. miliaceum*. This is especially critical for the plants growing at the low fertility conditions of the tailings (lower available-P and δ^15^N at bare samples). Nitrogen-rich soils, such as that of the control area, may provide suitable environmental conditions for its fast biogeochemical transformations leading to an enrichment of δ^15^N in soil and plants because most of steps in the N cycle discriminate against δ^15^N (Craine et al., 2009). By contrast, in nutrient-poor soils, such as that at the tailings, the lower discrimination against δ^15^N leads to lower soil and plant δ^15^N values (Ruiz-Navarro et al., 2016). The leaves of the plants at the tailings showed lower δ^15^N values than those at the control area but with similar total N foliar contents (~ 19 mg kg^−1^), which revealed the great efficiency of *P. miliaceum* for uptaking N under the low fertility conditions of the tailings (much lower soil δ^15^N values than at the control area). Previous pot experiments have shown the higher ability of *P. miliaceum* to compete for N compared to other species in mine tailings substrates (Martínez-Oró et al., 2017). This great ability to maximize nutrient acquisition under low available systems is a key factor for favouring plants’ environmental plasticity. The lower leaf δ^13^C values found at the tailings compared to the control site may indicate lower water use efficiency (WUE) of the tailings` plants. The lower competition for water resources due to the lower density of plant cover could have favoured a higher leaf stomatal conductance and transpiration of the plants at the tailings compared to those at the control site (Moreno-Gutiérrez et al., 2011).

**Table 2:** Parameters for plant leaf samples taken from the tailings pile and the control site

		Tailings pile		Control
*Elemental concentrations*				
C	g kg^−1^	408 ± 8		428 ± 2
N		15 ± 1		19 ± 1
P	mg kg^−1^	443 ± 24		365 ± 32
S		3034 ± 184	*	1336 ± 91
*Isotopic composition*				
δ^13^C	‰	− 30.22 ± 0.13	*	− 28.47 ± 0.16
δ^15^N		− 4.50 ± 1.25	*	6.42 ± 0.66
*Metal concentrations*				
Cu	mg kg^−1^	4.2 ± 0.4	*	2.7 ± 0.2
Mn		103 ± 14	*	43 ± 7
Pb		4.7 ± 0.5	*	1.0 ± 0.3
Zn		88 ± 18		31 ± 4

Data are average ± standard error. Number of replicates is three

*Between columns represents significant differences (*t*-test, *p* < 0.05) between columns

### Bacterial composition

The comparison among phyla bacterial composition may serve as a general approach to describe the effects of environmental parameters in microorganisms. First, we compare bare tailings to *P. miliaceum* rhizosphere tailings samples. With this comparison, we expect to evaluate the effect of the presence of *P. miliaceum* in the microbial successional processes at tailings. Then, we compare the results of *P. miliaceum* rhizospheres between the tailings and the control site to evaluate whether the tailings rhizosphere’s plants mimic the microbiome of the not-impacted sites.

**Fig. 2:**
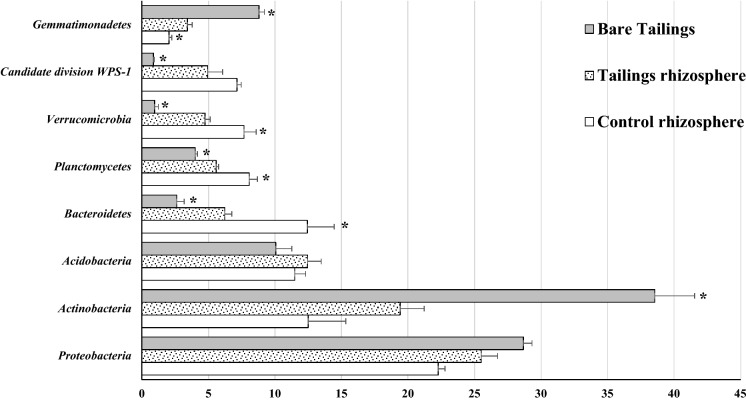
Percentage of bacterial phyla abundance for each sample. Data are average of three replicates. Bars on columns are standard error. *On columns represents significant differences (*t* test, *p* < 0.05) in relation to tailings rhizospheres samples

**Fig. 3:**
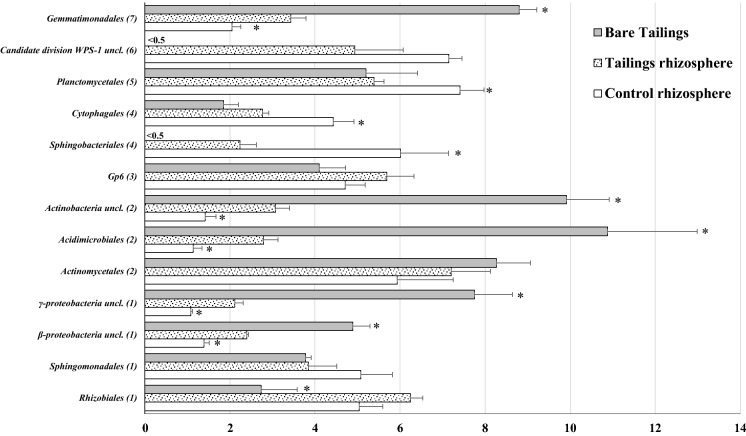
Percentage of bacterial order abundance for each sample. Data are average of three replicates. Bars on columns are standard error. *On columns represents significant differences (*t* test, *p* < 0.05) in relation to tailings rhizospheres samples. (1) belong to phylum *Proteobacteria*; (2) belong to phylum *Actinobacteria*; (3) belong to phylum *Acidobacteria*; (4) belong to phylum *Bacteroidetes*; (5) belong to phylum *Planctomycetes*; (6) belong to phylum *Candidate division WPS-1*; (7) belong to phylum *Gemmatimonadetes*

The edaphic parameters of the bare samples may condition the development of typical bacteria colonizers at early successional edaphic stages (Colin et al., 2019). The bare tailings samples were dominated by two bacterial phyla, *Proteobacteria* and *Actinobacteria*, which accounted for more than 50% of all bacteria (Fig. 2). These two groups are known to include specific lithotrophic taxa with a good ability to proliferate in metal-enriched environments (Wakelin et al., 2012). For instance, *γ-proteobacteria* uncl. and *β-proteobacteria* uncl. (Fig. 3) were the main order contributors (*p* < 0.05) within the *Proteobacteria* phylum, while *Acidimicrobiales* and *Actinobacteria* uncl. were the main orders (*p* < 0.05) within the *Actinobacteria* phylum. All these orders are known to dominate barren materials due to their antagonism with plants (e.g. taxa orders of *γ-proteobacteria* class) or their affinity to grow in Sulphur-enriched environments (e.g. *Acidimicrobiales*) (Sun et al., 2018). The order *Gemmatimonadales* belonging to the *Gemmatimonadetes* phylum exhibited a similar behaviour, with higher relative abundance percentages (*p* < 0.05) at the bare samples than at the rhizosphere tailings samples. Taxa belonging to this order have been already shown to proliferate under metal-enriched neutral pH soils (Hu et al., 2021).

The *P. miliaceum* rhizosphere tailings samples showed higher DOC and microbiological indicators (MBC, enzymatic activities) than the bare tailings samples. This slight fertility improvement under similar total metal(loid) concentrations could have promoted the transition from a lithotrophic bacterial composition to a more oriented organotrophic structure (Risueño et al., 2020). Compared to the bare samples, the rhizosphere samples at tailings showed lower relative abundance percentages (*p* < 0.05) of those lithotrophic taxa belonging to the *Actinobacteria* phylum (e.g. *Acidimicrobiales*). Although the relative abundance percentages of the *Proteobacteria* phylum were similar between bare and rhizospheric samples, the contribution of orders was different. Compared to the bare samples, the rhizosphere tailings samples showed lower percentages of the chemoautotrophic taxa *γ-proteobacteria* uncl. and *β-proteobacteria* uncl. and a higher percentage of *Rhizobiales*. The latter is known to be related to plant rhizosphere development and is especially critical in metal polluted or highly saline soils where it may contribute to plant establishment favouring N fixation and plant nutrient acquisition (Etesami & Alikhani, 2019; Hao et al., 2014). This may explain the comparable concentrations of N and P obtained in leaf samples between plants at the tailings and the control site and supports previous findings on the strong competitive behaviour of *P. miliaceum* for nutrient acquisition (Martínez-Oró et al., 2017). The phyla *Bacteroidetes* and *Candidate division WPS-1,* whose relative abundances were also higher at the tailings rhizosphere samples, may include taxa which are promoted in the presence of organic matter. *Candidate division WPS-1* is a phylum associated with semiarid environments (Bona et al., 2021) which is favoured by soil fertile conditions (Pan et al., 2021). The phylum *Bacteroidetes* included the orders *Cytophagales* and *Sphingobacteriales*, which have been shown to play an important role in the decomposition of organic matter under neutral pH environments (Reichenbach, 2006). Given the poor N (low total N and δ^15^N) and low available-P of the bare tailings samples, the presence within the *P. miliaceum* rhizosphere of these organotrophic taxa related to the cycling of C and N may indicate a step forward in the restoration of the edaphic functionality (Xiao et al., 2019).

Compared to the rhizosphere samples of the tailings, the bacterial composition of the control samples showed no significant differences in the percentages of the phyla *Actinobacteria* and *Proteobacteria*. However, the significant differences took place at order level. For instance, the control samples showed lower relative abundance percentages (*p* < 0.05) of the lithotrophic *Acidimicrobiales, γ-proteobacteria* uncl. and *β-proteobacteria* uncl. corroborating the low competition character of these groups in fertile vegetated soils (Sun et al., 2018). The lower high metal concentration and better soil fertility indicators of the control samples allowed the occurrence of higher relative abundance percentage of taxa related to the decomposition of organic matter such as *Cytophagales* and *Sphingobacteriales* (both belonging to the *Bacteroidetes* phylum) (Reichenbach, 2006) or those which are sensitive to high metal concentrations such as *Planctomycetales* (belonging to the *Planctomycetes* phylum) (Fuerst & Sagulenko, 2011).

### Fungal composition

**Fig. 4:**
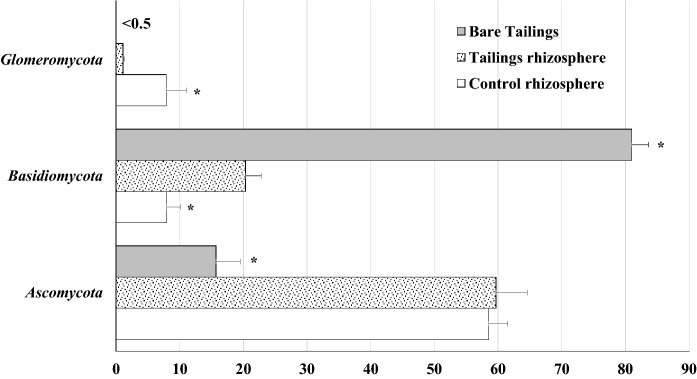
Percentage of fungal phyla abundance for each sample. Data are average of three replicates. Bars on columns are standard error. *On columns represents significant differences (*t* test, *p* < 0.05) in relation to tailings rhizospheres samples

Fungal composition showed different patterns when comparing bare tailings soil samples and the *P. miliaceum* rhizosphere samples (both, at the tailings and control site). While *Basidiomycota* was the main phylum for the bare tailings samples, *Ascomycota* appeared as the main phylum for the rhizosphere samples (both, tailings and control) (Fig. 4). This contrasting behaviour of both fungal phyla could be explained by their well-known antagonism (Egidi et al., 2019) and selective preference for specific organic matter compounds (Ma et al., 2013).

**Fig. 5:**
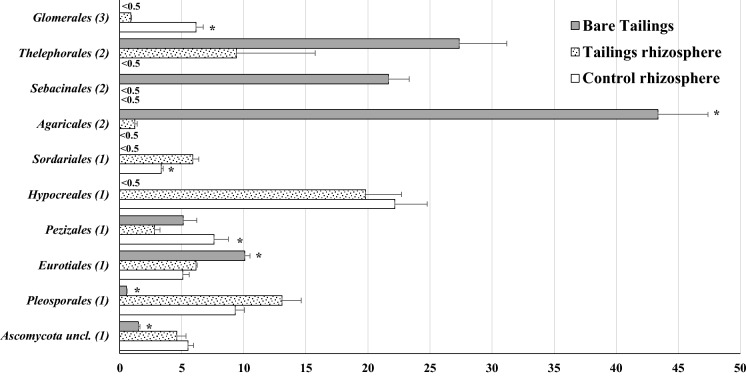
Percentage of fungal order abundance for each sample. Data are average of three replicates. Bars on columns are standard error. *On columns represents significant differences (*t* test, *p* < 0.05) in relation to tailings rhizospheres samples. (1) belong to phylum *Ascomycota*; (2) belong to phylum *Basidiomycota*; (3) belong to phylum *Glomeromycota*

The *Basidiomycota* phylum showed around 80% of the relative abundance in bare tailings samples. This was mainly due to the contribution of the orders *Thelephorale*s, *Sebacinales* and *Agaricales* (Fig. 5). Some of these taxa, such as *Thelephorales*, have shown high tolerance to abiotic stress such as salinity (Thiem et al., 2018). The *Ascomycota* phylum showed around 60% of the relative abundance percentages in the *P. miliaceum* rhizosphere (both at the tailings and the control), mainly due to the contribution of *Hypocreales* (20%) and *Pleosporales* (10–15%). These two orders have been also identified in the rhizospheres of two ruderal plant species adapted to saline soils, such as *Zygophyllum fabago* (Risueño et al., 2020) and *Hordeum vulgare* (Murphy et al., 2015). Interestingly, rhizospheres from the two locations of our study also showed similar relative abundance data (~ 5%) for *Ascomycota uncl., Eurotiales* and *Sordariales*. The specific biogeochemical processes at the plants rhizospheres of *P. miliaceum* are conditioned by their specific exudates which in turn may reconfigure a specific microbiome (Colin et al., 2017).

## Conclusions

The comparison of the rhizosphere edaphic parameters and leaf elemental composition of *P. miliaceum* plants growing at a mine tailings pile and a dry crop land site (not polluted) revealed that, in spite of the contrasting edaphic fertility conditions of both sites, essential nutrients such as N and P were in similar concentrations for leaf samples. Obviating some lithotrophic bacterial taxa found at the tailings, which may not play a significant role in organic matter cycling, those organotrophic bacteria, whose relative abundance percentages were higher in tailings rhizospheres compared to bare tailings samples, were also found in the rhizosphere of the control site (e.g. *Cytophagales, Sphingobacteriales, Rhizobiales* or *Candidate division WPS-1* uncl). The presence at the tailings rhizospheres of bacterial orders, such as *Rhizobiales,* related to the cycling of N and that may facilitate its acquisition by plants, could explain the comparable N leaf concentrations between sampling sites. For the case of fungal composition, the rhizospheres from both locations also shared a relevant proportion of fungal orders. This may indicate that *P. miliaceum* is able to shape its own specific microbiome at the tailings independently from the initial microbial tailings composition.

### Supplementary Information

Below is the link to the electronic supplementary material.

Supplementary file 1 (PDF 496 kb)

## Data Availability

Not applicable.
