# Supplementary material for: Evaluation of the rhizospheric microbiome of the native colonizer Piptatherum miliaceum in semiarid mine tailings
Source: Environ Geochem Health. 2022 Sep 8;45(12):9359–71. doi: 10.1007/s10653-022-01357-y (PMC10673988; doi:10.1007/s10653-022-01357-y)
Supplement: Supplementary file 1 — (PDF 496 kb) [file 10653_2022_1357_MOESM1_ESM.pdf]

Supplementary Material for

Evaluation of the rhizospheric microbiome of the native colonizer *Piptatherum miliaceum* in semiarid mine tailings

Héctor M. Conesa[1]*, Isabel Párraga-Aguado[1], Francisco J. Jiménez-Cárceles[2], Yolanda Risueño[1]

Héctor M. Conesa[1*]

Universidad Politécnica de Cartagena, Escuela Técnica Superior de Ingeniería Agronómica Departamento de Ingeniería Agronómica. Paseo Alfonso XIII, 48. 30203 Cartagena (Spain)

e-mail: hector.conesa@upct.es

ORCID: 0000-0003-0747-3401

*corresponding author

Isabel Párraga-Aguado[1]

Cartagena (Spain)

e-mail: parraga.isabel@gmail.com

ORCID: 0000-0002-3199-9342

Francisco J. Jiménez-Cárceles[2]

BIOCYMA, Consultora en Medio Ambiente y Calidad, S.L.

C. Azarbe del Papel, 10, 30007 Murcia (Spain)

e-mail: francisco@biocyma.com

ORCID: 0000-0001-7119-9991

Yolanda Risueño[1]

Cartagena (Spain)

e-mail: yolandarg814@gmail.com

ORCID: 0000-0002-6204-6210

This supplementary material contains extended protocols and three Figures.

Extended protocols for DNA extraction, PCR amplification and sequencing

Microbial (bacteria and fungi) DNA was extracted from 0.25g soil using the PowerSoil DNA Isolation Kit (MOBIO), according to the manufacturer's instructions. The isolated DNA was quantified using a NanoDrop 2000 spectrophotometer. Library preparation and Illumina sequencing were carried out at the IPBLN Genomics Facility (CSIC, Granada, Spain). Amplicon libraries targeting the 16S rRNA gene and ITS2 region were generated by a two-steps PCR strategy. Gene-specific amplification was performed in triplicate with 15ng of soil-extracted DNA in a final volume of 10 µl. Gene specific primers, V3V4fw (5′ CCTACGGGNGGCWGCAG 3′), V3V4rev (5′ GACTACHVGGGTATCTAATCC 3'), ITS3_KYO2-Fw (5′ GATGAAGAACGYAGYRAA 3') and ITS4-Rev (5′ TCCTCCGCTTATTGATATGC 3'), were designed with Nextera overhang adapters. Primers were used at a final concentration of 0.2 µM. Reaction was performed with 1x KAPA HiFi Hot Start Ready Mix DNA polymerase (Roche Diagnostics, West Sussex, United Kingdom). Cycling conditions were 95°C for 3 min; 25 x (95°C for 30 s, 55°C for 30 s, 72°C for 30 s) and then 72°C for 5 min for 16S amplification and 95°C for 3 min; 27 x (95°C for 30 s, 47°C for 30 s, 72°C for 30 s) and then 72°C for 5 min for ITS2 amplification. Triplicates were pooled together and validated through visualization on a 1.8% (w/v) agarose gel. Amplicons were then purified using NucleoMag® NGS Clean-up and Size Select Kit (Macherey-Nagel, Düren, Germany). A second PCR step attached dual combinatorial indices and Illumina sequencing adapters using Nextera XT v2 index kit. Cycling conditions were 95°C for 3 min, 8 x (95°C for 30 s, 55°C for 30 s, 72°C for 30 s) and then 72°C for 5 min. Amplicon generation was validated again through visualization on a 1.8% (w/v) agarose gel and cleaned with NucleoMag® NGS Clean-up and Size Select Kit (Macherey-Nagel). Concentration was measured on the Qubit® fluorometer (Thermo). Amplicons were pooled in an equimolecular manner and final library mix was run on a Bioanalyzer HS DNA chip to verify quality and size distribution. The library pool was then diluted and denatured as recommended by Illumina MiSeq library preparation guide. The 300x2nt paired-end sequencing was conducted on a MiSeq sequencer.

Bioinformatics and statistical analysis

Raw sequence data in FASTQ format (16S and ITS2) were subjected to quality control analysis with FastQC software and prepared for taxonomic classification using the Mothur software (version 1.43.0) (Schloss et al., 2009) and following the standard operating protocol proposed by (Kozich et al., 2013). Overlapping pairs of sequence reads were merged into contigs. In addition, reads with ambiguous bases, duplicated contigs and homopolymers longer than 13 bp were removed. The VSEARCH algorithm (embedded in the Mothur framework) was used to remove chimeras and these were subsequently omitted. The resulting sequences were classified according to the taxonomy into the corresponding Operational Taxonomic Units at 97% similarity, besides using the reference trainset 16_022016.pds from https://mothur.org/wiki/RDP_reference_files for Bacteria and ITS sequences provided by the UNITE ITS database (version 7.2) at https://unite.ut.ee/repository.php for Fungi. Undesired lineages such as Plantae, Animalia, Protista, "unknown" and other were removed. The final sequences were then grouped into taxonomic groups (phylum and order), using the phylotype command in Mothur, which relies upon reference taxonomic outlines to classify sequences to taxonomic bins (Schloss and Westcott, 2011). Relative abundances of different taxonomic levels of each bacterial and fungal group were calculated as the percentage from the total count of reads in each sample using the get.relabund command in Mothur. Phyla and orders (both bacteria and fungi) that showed >5% relative abundance in at least one sampling site, were considered. The taxa with <0.5% of relative abundance were discarded for comparison.

References

Kozich, J.J., Westcott, S.L., Baxter, N.T., Highlander, S.K., Schloss, P.D., 2013. Development of a Dual-Index Sequencing Strategy and Curation Pipeline for Analyzing Amplicon Sequence Data on the MiSeq Illumina Sequencing Platform. Appl. Environ. Microbiol. 79, 5112–5120. https://doi.org/10.1128/aem.01043-13

Schloss, P.D., Westcott, S.L., 2011. Assessing and improving methods used in operational taxonomic unit-based approaches for 16S rRNA gene sequence analysis. Appl. Environ. Microbiol. 77, 3219–3226. https://doi.org/10.1128/AEM.02810-10

Schloss, P.D., Westcott, S.L., Ryabin, T., Hall, J.R., Hartmann, M., Hollister, E.B., Lesniewski, R.A., Oakley, B.B., Parks, D.H., Robinson, C.J., Sahl, J.W., Stres, B., Thallinger, G.G., Van Horn, D.J., Weber, C.F., 2009. Introducing mothur: Open-Source, Platform-Independent, Community-Supported Software for Describing and Comparing Microbial Communities. Appl. Environ. Microbiol. 75, 7537–7541. https://doi.org/10.1128/AEM.01541-09

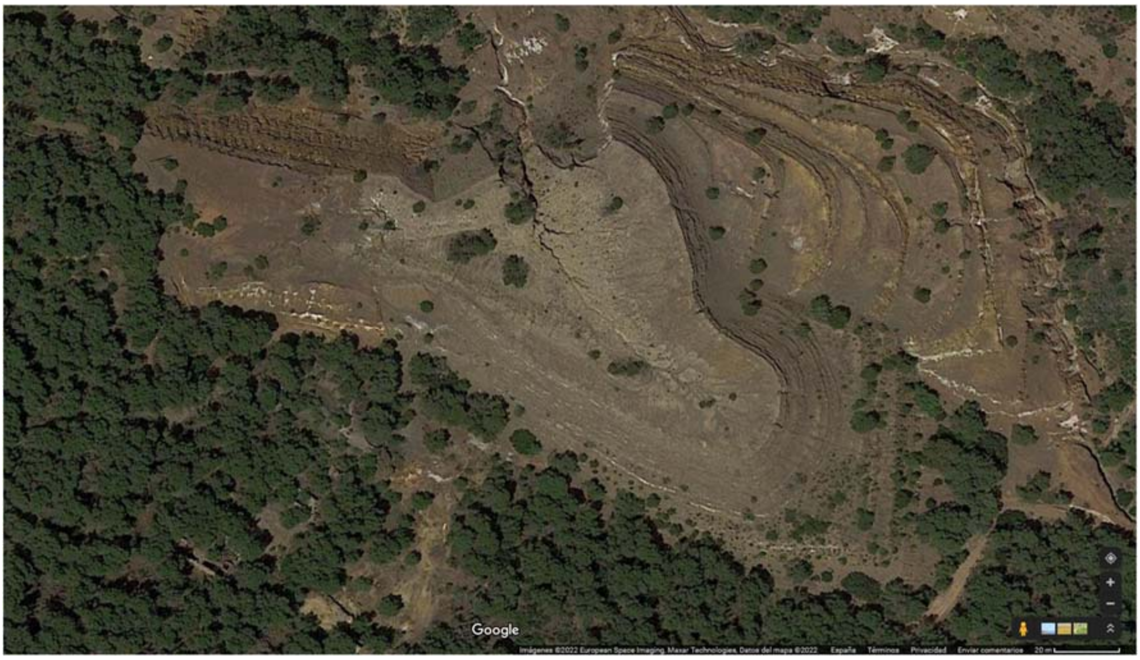

Figure SM1-Aereal view of the tailings pile. Adapted from Google Maps. Online aerial view. Visited, 10th June 2022. Recovered from https://www.google.es/maps/@37.6039937,-0.8345852,219m/data=!3m1!1e3?hl=es

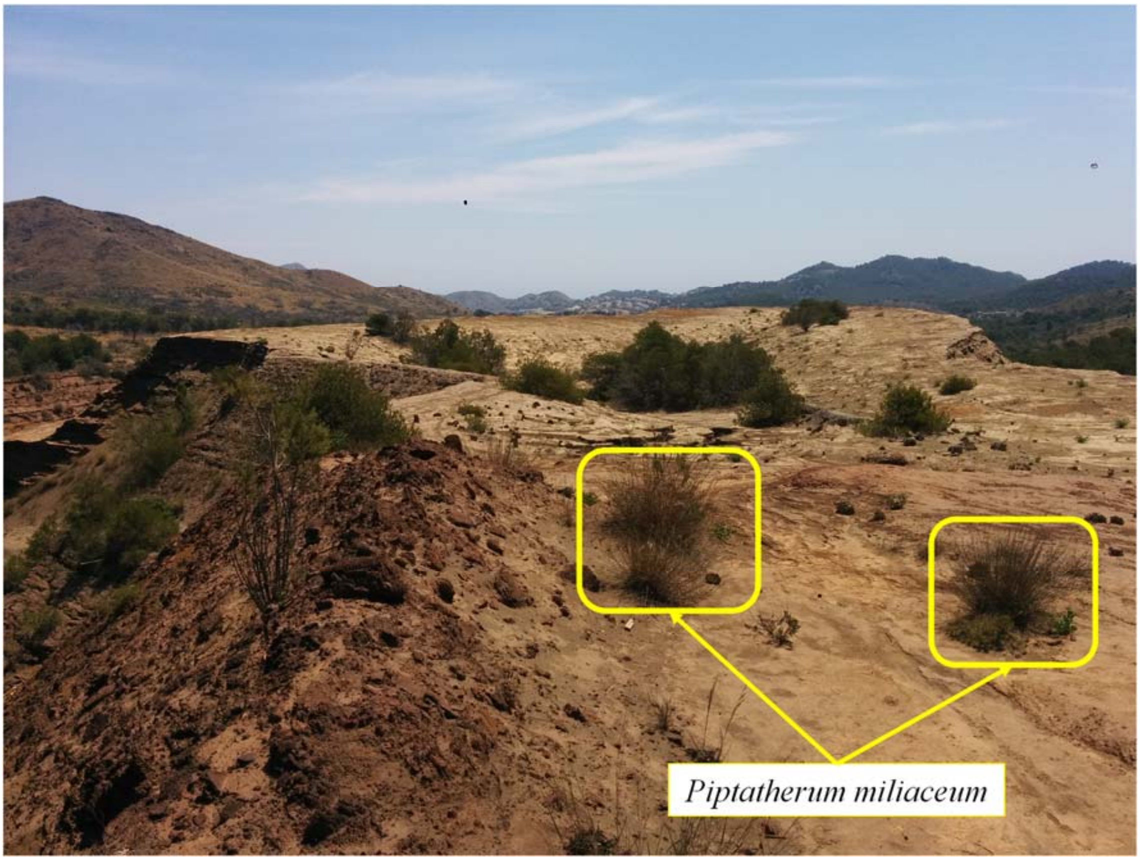

Figure SM2-View of the tailings pile and some individuals of *Piptatherum miliaceum*.

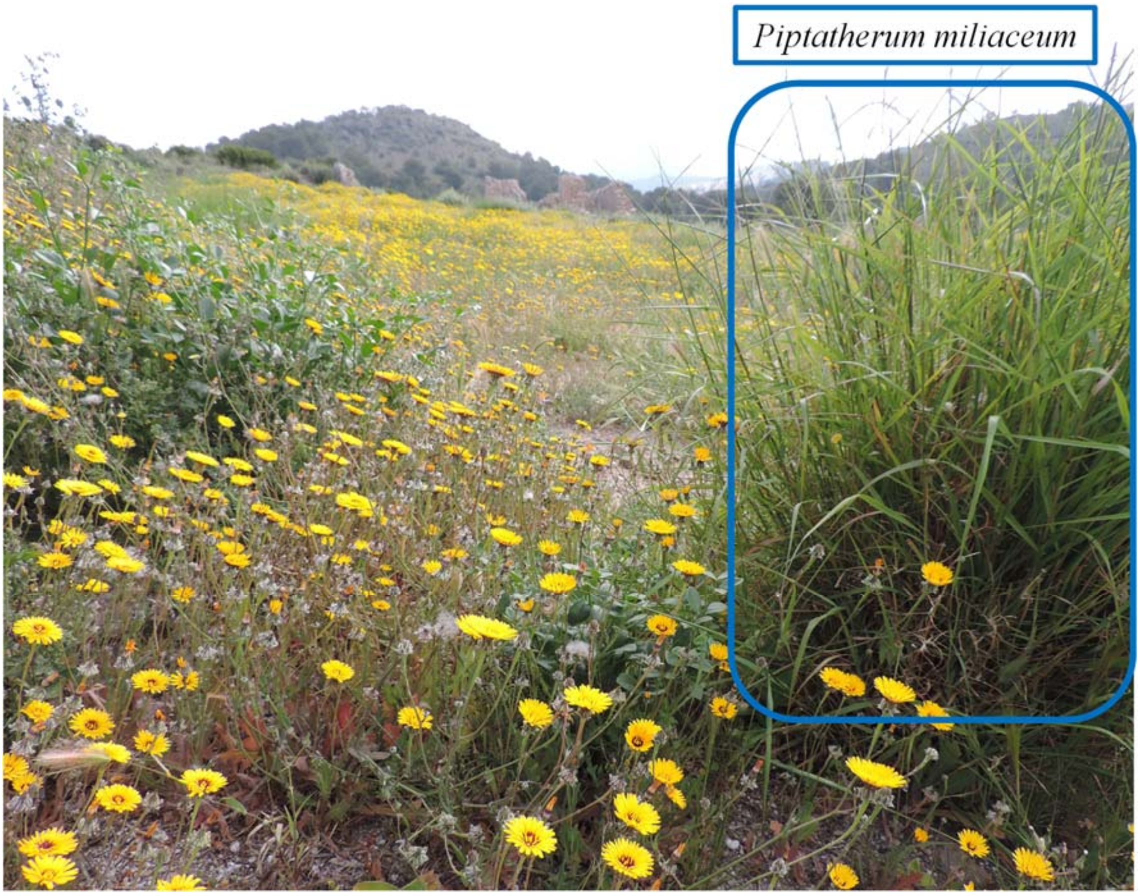

Figure SM3-View of the control site area and an individual of *Piptatherum miliaceum*.
